# Effect of Exogenous Factors on Bacteriocin Production from *Lactobacillus paracasei* J23 by Using a Resting Cell System

**DOI:** 10.3390/ijms141224355

**Published:** 2013-12-13

**Authors:** Huaxi Yi, Xue Han, Yanyan Yang, Wenli Liu, Hui Liu, Yingchun Zhang, Kai Sun, Lanwei Zhang, Fang Ma

**Affiliations:** 1 School of Food Science and Engineering, Harbin Institute of Technology, Harbin 150090, China; E-Mails: yihuaxi@hit.edu.cn (H.Y.); xhan@hit.edu.cn (X.H.); yyyanghit@yeah.net (Y.Y.); liuwenlihit@yeah.net (W.L.); liuhuihit@yeah.net (H.L.); zhangyingchun@hit.edu.cn (Y.Z.); wangzhuan@hit.edu.cn (K.S.); 2 School of Municipal and Environmental Engineering, Harbin Institute of Technology, Harbin 150090, China; E-Mail: mafang@hit.edu.cn

**Keywords:** lactic acid bacteria, bacteriocin, *Lactobacillus paracasei*, resting cell, system, exogenous factors

## Abstract

A resting cell system was developed for bacteriocin Lac-B23 production from *Lactobacillus paracasei* J23. The resting cell medium contained (g/L): Glucose 20, Sodium acetate 5.0, MnSO_4_ 0.25 MgSO_4_ 0.5, Ammoniumhydrogencitrate 1.0, KH_2_PO_4_ 1.0. The resting cell incubation time and temperature were 20 h and 37 °C and the effects of exogenous factors, including amino acids, glycerol, pyruvic acid, and α-ketoglutaric acid were investigated. Cys and Gly could stimulate the production of bacteriocin, while no stimulus effect was observed for Glu, Tyr and Ala. Glycerol and pyruvic acid increased bacteriocin production and the optimum concentrations were 1% and 30 g/L, respectively. Bacteriocin could act as an inducer of its own biosynthesis. These findings are of importance for the further study of bacteriocin biosynthesis regulation and for the improvement of bacteriocin production yields.

## 1. Introduction

Bacteriocins are ribosomally synthesized antibacterial proteins produced by different bacterial species [1–3]. Bacteriocins produced by lactic acid bacteria (LAB) are attractive to the food industry because of their potential as natural food preservatives, and are generally recognized as safe (GRAS) [4,5]. In the last decades, a variety of LAB bacteriocins have been identified and characterized [6,7]. New bacteriocins are still being discovered and developed. However, only Nisin has achieved a commercial scale successfully and it is used as a food preservative worldwide [8]. A potential bottleneck in LAB bacteriocin commercialization could be low production yields. Therefore, it is very important to enhance bacteriocin production. It has been observed that the production of bacteriocin is influenced by the bacterial growth phase [9,10], medium composition [11–14], and culture conditions [15–18]. In addition, bacteriocin production can be induced by exogenous factors or exhibit auto-induction [19–21]. It is reasonable to speculate that LAB bacteriocin production could also be induced by bacteriocin precursors or intermediate metabolites of the bacteriocin synthesis pathways. Presently, little information is available about the effect of such exogenous factors on the bacteriocin production, such as amino acids, glycerol, pyruvic acid, α-ketoglutaric acid, and others.

LAB growth and bacteriocin production are strongly influenced by carbon sources, nitrogen sources, growth factors, and other exogenous factors. The traditional technique to optimize the above multivariable system is “one-factor at a time” with growing cultures. It may result in incorrect conclusions since effects on cell growth are not readily differentiated from effects on bacteriocin production. The resting cell technique can eliminate many of the problems associated with studies made on growing cultures. Resting cells are non-proliferating and regarded as a source of enzymes for studies on bacterial metabolism. A resting cell system provides a useful tool for studying the regulation of metabolite synthesis, particularly for the influence of factors on bacterial metabolism. Resting cell technology has been successfully applied in many areas of biotechnology such as optimization of antibiotic production [22], amino acid metabolism [23], nisin biosynthesis [24], and exotoxin production [25], but it has not yet been reported as a means to study and optimize conditions of a growth medium and to screen for the effects of exogenous factors on bacteriocin production.

In the previous study, *Lactobacillus paracasei* J23 isolated from Chinese traditional fermented vegetable juice could produce bacteriocin Lac-B23 with a broad inhibitory spectrum [26]. In this paper, to facilitate the bacteriocin biosynthesis of *Lactobacillus paracasei* J23, a resting cell system was developed and used to select the exogenous factors affecting production of the LAB bacteriocin. The objectives of this work were to evaluate the effects of the exogenous factors on bacteriocin production, and to identify exogenous inducers able to improve bacteriocin yield.

## 2. Results and Discussion

### 2.1. Establishment of the Resting Cell System

The component of candidate culture medium for the resting cell system is based on the common MRS medium composition. In previous preliminary studies, the composition of MRS has been optimized in preparation for a resting cell system [27]. Based on those studies, the resting cell system was developed as shown in Table 1.

It was observed that the antimicrobial activity was 160U while the nucleic acid content changed little in **#**5 culture medium. The results showed that the cells of *Lactobacillus paracasei* J23 maintained the resting state and did not proliferate in **#**5 medium. It is also found though *Lactobacillus paracasei* J23 grew better in **#**2 medium than in **#**5 medium, but the antimicrobial activity is the same with that of the #5 medium, which suggested that higher biomass concentration could not necessarily result in higher bacteriocin production.

**Figure 1.**
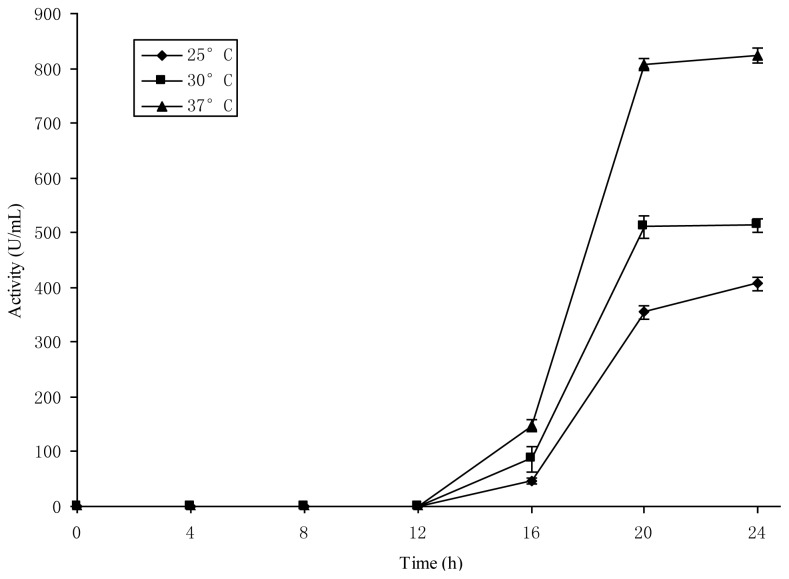
Effect of time and temperature on Lac-B23 production from *Lactobacillus paracasei* J23 in resting cell medium at 25 °C, 30 °C, 37 °C for 24 h, respectively. The composition of the resting cell medium is shown in Table 1. Values are expressed as mean value and standard deviation of triplicate determinations. Mean values (*n* = 3) for each experiment are presented.

To validate the #5 culture medium as a resting cell system, 0.75% NaCl solution was used as control to reduce the bacteriocin Lac-B23 from the inoculation. The results in Table 2 showed that *Lactobacillus paracasei* J23 could produce bacteriocin under resting cell state in #5 culture medium. It was indicated that the #5 culture medium met the criteria of a resting cell system based on the high antimicrobial activity and little ΔNucleic acid, and was confirmed as a resting cell system. The optimum culture time and temperature were 20 h and 37 °C (Figure 1). Therefore, the #5 culture medium was appropriate as a resting cell system and used to screen potential exogenous inducers for bacteriocin Lac-B23 production.

At present, the response surface methodology (RSM) has been used to optimize medium components [28]. RSM was used to optimize the resting cell medium in a previous study [29], but it was found that the results of RSM in the laboratory were not useful in a pilot scale test. It was also found that though the conventional “one-at-a-time-approach” is cumbersome and time consuming, it does lead to a substantial increase in Lac-B23 production: The results of the resting cell medium optimization by using “one-at-a-time-approach” are also reproducible in the pilot scale test and therefore, was selected to optimize the resting cell system in this study.

### 2.2. Effects of Amino Acid

**Figure 2.**
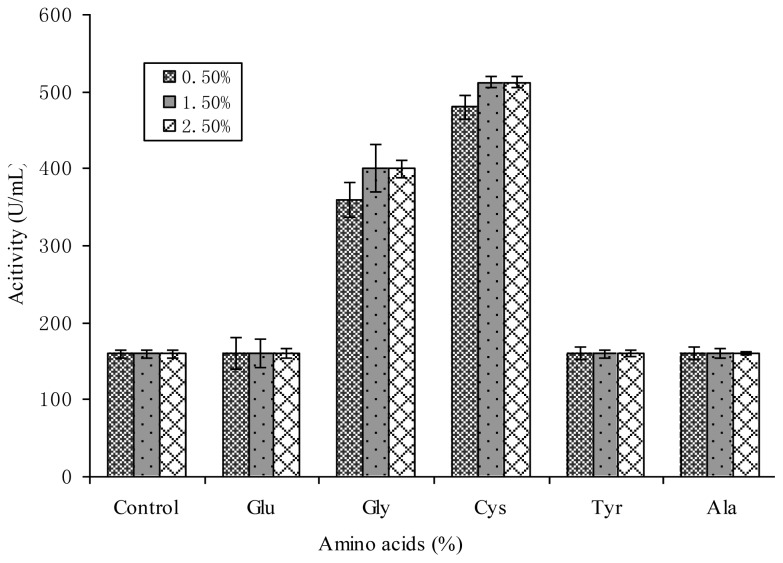
Effects of 5 amino acids addition on Lac-B23 production from *Lactobacillus paracasei* J23 in the resting cell medium at 37 °C for 20 h. The Lac-B23 production in the #5 medium was used as control. Values are expressed as mean value and standard deviation of triplicate determinations. Mean values (*n* = 3) for each experiment are presented.

According to previous study about the composition of amino acids in the bacteriocin Lac-B23 [30], the effects of Glu, Gly, Cys, Tyr and Ala with various concentration on bacteriocin Lac-B23 production were investigated in the established resting cell system (Figure 2).

The results in Figure 2 show that the bacteriocin Lac-B23 production was stimulated by Gly and Cys, and that the antimicrobial activity was increased from 160 U to 400 U (Gly) and 520 U (Cys) when the added concentration was above 1.5%. Though Glu, Tyr and Ala were not found to increase bacteriocin Lac-B23 production, it was observed they could stimulate the growth of *Lactobacillus paracasei* J23. It was speculated that the positive effects of Glu, Tyr and Ala on cell growth could be due to their roles as nitrogen sources or growth factors. In addition, Gly and Cys could promote the bacteriocin Lac-B23 biosynthesis as precursor. The effects of amino acids on nisin and pediocin productions by two lactic acid bacteria have been reported [31], and the similar phenomenon was observed. Detailed knowledge about the fundamental mechanisms of the above two amino acid underlying the regulation of bacteriocin production is not yet known.

### 2.3. Effects of Glycerol

**Figure 3.**
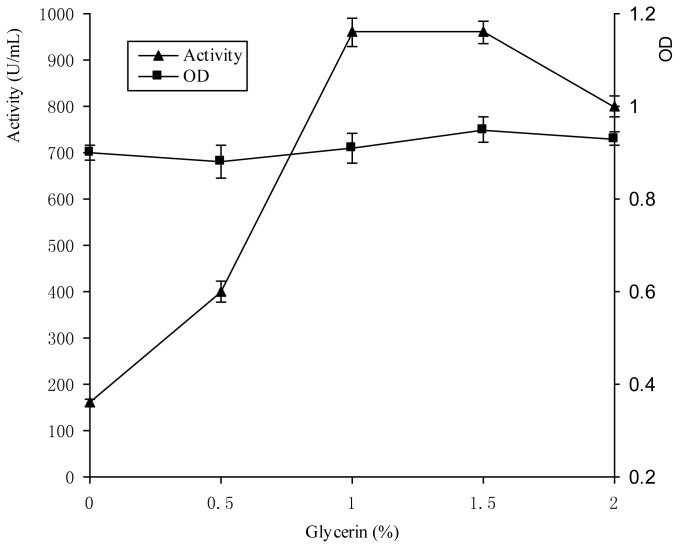
Effect of different addition of Glycerin on Lac-B23 production and *Lactobacillus paracasei* J23 growth in resting cell medium. Values are expressed as mean value and standard deviation of triplicate determinations. Mean values (*n* = 3) for each experiment are presented.

Glycerol is an important intermediate in the metabolic pathways, which can regulate the organism metabolism. The effects of glycerol on bacteriocin Lac-B23 production were investigated in the established resting cell system (Figure 3).

It showed that glycerol could promote bacteriocin Lac-B23 production. When the added glycerol concentration is above 1%, the bacteriocin Lac-B23 reached a stationary state. Though glycerol was involved in Embden-Meyerhof-Parnas pathway (EMP) and could be used as energy for cell growth theoretically, the *Lactobacillus paracasei* J23 kept the resting cell state in the whole process. Therefore, it was reasonable that the improvement of bacteriocin Lac-B23 production was not attributed to the cell growth, and glycerol could be added as positive inducer in bacteriocin Lac-B23 production.

### 2.4. Effects of Pyruvic Acid

**Figure 4.**
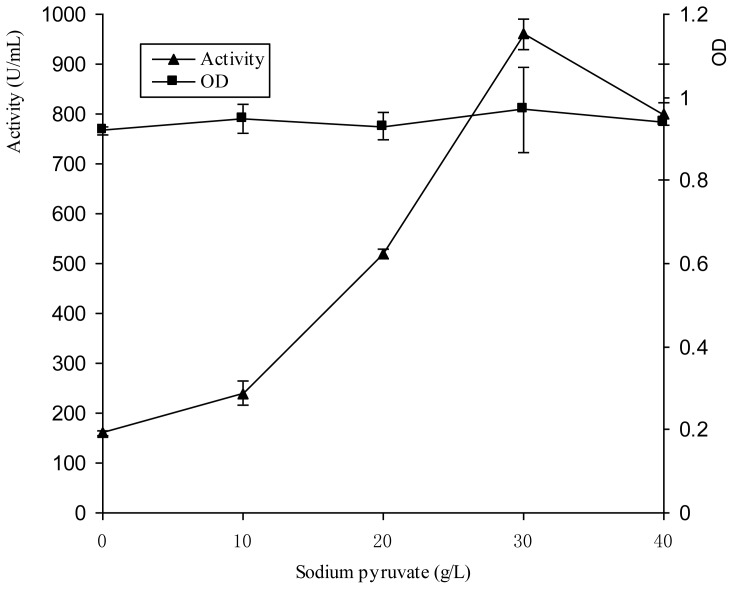
Effect of Sodium pyruvate on Lac-B23 production and *Lactobacillus paracasei* J23 growth in the resting cell medium. Values are expressed as mean value and standard deviation of triplicate determinations. Mean values (*n* = 3) for each experiment are presented.

Pyruvic acid is regarded as one of the key intermediates in Embden–Meyerhof–Parnas (EMP) pathways and the tricarboxylic acid (TCA) cycle, and play significant roles in protein, peptide and amino acid biosynthesis. The effects of various concentration of pyruvic acid on bacteriocin production were studied in the established resting cell system (Figure 4).

The results show that pyruvic acid exhibits similar effects as glycerol on bacteriocin Lac-B23 production. The optimum concentration was 30g/L, and the *Lactobacillus paracasei* J23 cells remained in the resting cell state during the fermentation. It was interesting that the pH was observed to decrease from 6.5 to 5.5 quickly. It was assumed that pyruvic acid was used as the precursor to promote the biosynthesis of lactic acid, thus leading to the pH decreased. In our previous study, pH had a significant effect on the bacteriocin production, which was enhanced by the relatively low pH. The optimum pH for bacteriocin Lac-B23 production was 5.5. Therefore, it was thought that the effect of glycerol on bacteriocin Lac-B23 production was indirect. It was indicated that addition of pyruvic acid might initiate the bacteriocin Lac-B23 biosynthesis earlier or extend the effective biosynthesis stage.

### 2.5. Effects of Bacteriocin as Self-Inducer

**Figure 5.**
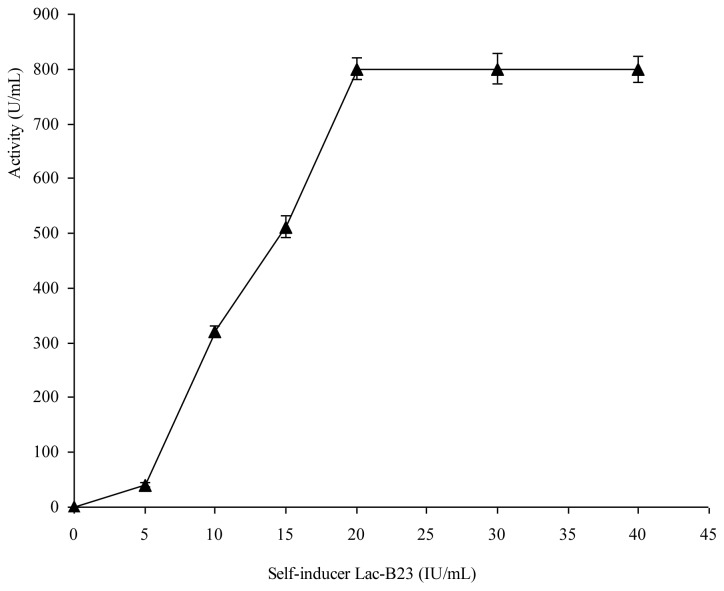
Dose-dependent of Lac-B23 production induced by itself from *Lactobacillus paracasei* J23. Values are expressed as mean value and standard deviation of triplicate determinations. Mean values (*n* = 3) for each experiment are presented.

**Figure 6.**
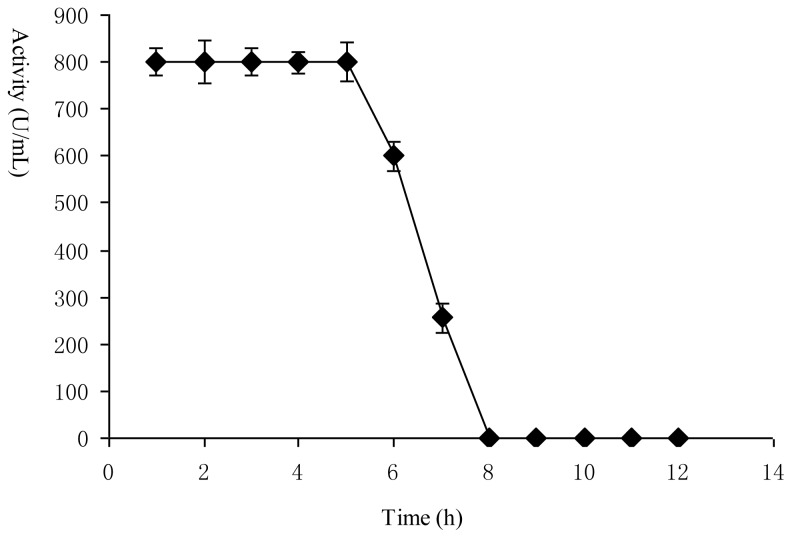
Time-dependent of Lac-B23 production induced by itself from *Lactobacillus paracasei* J23. Values are expressed as mean value and standard deviation of triplicate determinations. Mean values (*n* = 3) for each experiment are presented.

It has been reported that bacteriocin biosynthesis can be dependent on the presence of an extracellular peptide of the bacteriocin itself produced by the strain, and that bacteriocin may act as an extracellular regulators of its own biosynthesis [19,20]. The effects of the added dose and time of Lac-B23 as inducer on its own production were investigated (Figures 5 and 6).

The results suggested that bacteriocin is able to induce ist own production in a dose- and time-dependent manner. the threshold of induction occurred at 20 U/mL, and temporal effectiveness assessments revealed that Lac-B23 must be added before stationary phase (before 6 h) when it was used as inducer. It is indicated that adding extracellular inducer peptide is necessary to achieve a high level of bacteriocin production. This is in agreement with the report of Kleerebezem [32]. Time-dependent control strategies for enhancing synthesis of bacteriocin have been studied by using artificial neural networks and genetic algorithms [33]. The optimization of dose-dependent and time-dependent of bacteriocin Lac-B23 is currently in progress. The fundamental regulation mechanisms have been identified through a quorum–sensing based pathway, composed of an induction peptide, a histidine protein kinase and a response regulator [34]. Further studies of the regulation of bacteriocin biosynthesis on the transcription level are required.

## 3. Experimental Section

### 3.1. Microorganisms

*Lactobacillus paracasei* J23, which is the bacteriocin Lac-B23 producer obtained from previous screening efforts, was used in this work. *L. monocytogenes* was used as the indicator organism in the bacteriocin activity assay.

### 3.2. Antimicrobial Activity Assay

The antagonistic activity was measured by the well-diffusion method. Bacteriocin sample were serially diluted two-fold, and the reciprocal of the highest inhibitory dilution was used to express the arbitrary activity units (AU) per milliliter. In order to eliminate the inhibition of lactic acid on the test organisms, the tested supernatants were adjusted to pH 6.0 with 1 M NaOH and treated with catalase (1 mg/mL) to exclude the inhibition due to hydrogen peroxide production.

### 3.3. Resting Cell System

Five different culture media (as shown in Table 1) were formulated based on MRS medium and our previous preliminary study. Fifty milliliters of different culture medium was inoculated with 1% of 24 h old *Lactobacillus paracasei* J23 culture and incubated at 37 °C for 24 h. The antagonistic activity, nucleic acid content (NAC) and dried cell weight (DCW) of *Lactobacillus paracasei* J23 were examined. The culture medium was confirmed as the resting cell system based on the strong antimicrobial activity, and little change in nucleic acid content. The effect of time and temperature on Lac-B23 production in the resting cell system was investigated at 25, 30, 37 °C for 24 h, respectively. The cell concentration was assayed with a spectrophotometer (Shanghai Spectrum Co. Ltd, Shangshai, China) at 600 nm.

### 3.4. Nucleic Acid Content

The *Lactobacillus paracasei* J23 cell suspension was centrifuged at 8000× *g* for 15 min, and the cell pellets were washed three times with trichloroacetic acid and ethanol, then centrifuged at 8000× *g* for 15 min and extraction with 5% trichloroacetic acid at 80 °C for 30 min. The supernatant was diluted and the optical density (OD 260 nm) was assayed with spectrophotometer. ΔNucleic acid (g/L) was measured as the difference of nucleic acid content between before and after resting cultures.

### 3.5. Exogenous Inducers Selection

The effects of amino acids (Glu, Gly, Cys, Tyr, Ala), glycerol, and pyruvic acid on bacteriocin production were investigated by using the established resting cell system. The above five amino acids (0.5%, 1.5%, 2.5%), glycerol (*v*/*v*, 0.5%, 1.0%, 1.5%, 2.0%), pyruvic acid (10 g/L, 20 g/L, 30 g/L, 40 g/L) were added to the resting cell system for the examination of the effects on the bacteriocin production, respectively. Values are expressed as mean value and standard deviation of triplicate determinations.

### 3.6. Self-Induction of Bacteriocin

The added dose (5 U/mL to 40 U/mL) and added time (0–12 h) of bacteriocin Lac-B23 as self-inducer were investigated in the established resting cell system.

### 3.7. Statistical Analysis

One-way ANOVA was applied to the result of technological properties. All experiments were performed 3 times, and the values are expressed as mean value and standard deviation of triplicate determinations. The SPSS software package (version 16.0, SPSS Inc., Chicago, IL, USA) was used for this purpose.

## 4. Conclusions

A resting cell system was developed and proved to be a useful tool in screening the exogenous factors on bacteriocin Lac-B23 production. It is possible to enhance yield of bacteriocin Lac-B23 by adding Cys, Gly, Glycerol, pyruvic acid and Lac-B23 itself. These findings are of importance for the further study of bacteriocin biosynthesis regulation and the improvement of bacteriocin yields. In addition, this report could advocate an interest again for the use of resting cell techniques in the areas of regulation and optimization of bacteriocin production.

## Figures and Tables

**Table 1.** Resting cell medium selection of *Lactobacillus paracasei* J23.

Ingredient (g/L)	Candidate culture medium for resting cell system				

	#1	#2	#3	#4	#5
Peptone	10	-	-	-	-
Beef extract	10	-	-	-	-
Yeast extract	5	5	-	-	-
Glucose	20	20	20	20	20
NaAC	5	5	5	5	5
Ammonium citrate dibasic	2	2	-	-	1
MnSO_4_	0.25	0.25	0.25	-	0.25
MgSO_4_	0.58	0.58	0.5	-	0.5
KH_2_PO_4_	2	2	-	-	1
Activity (U)	960	160	80	0	160
ΔNucleic acid (g/L)	2.14	1.89	−0.11	−0.20	0.05

**Table 2.** Validation test of resting cell system.

Medium	Before resting culture			After resting culture		

	DCW (g/L)	NAC (g/L)	Antimicrobial activity (U)	DCW (g/L)	NAC (g/L)	Antimicrobial activity (U)
#5	2.82	0.85	0	2.86	0.87	160
Control	2.79	0.76	0	2.71	0.69	0
